# A Thrombocytopenic Thrombotic Purpura in a Patient With a Metastatic HER2+ Breast Cancer: Description of a Case Report

**DOI:** 10.1002/ccr3.70395

**Published:** 2025-04-08

**Authors:** Raffaele Longo, Benjamin Savenkoff, Romane Ribal, Alexandre Dadoun, Marco Campitiello, Francesca Plastino, Pierre‐Olivier Legros, Nathalie Marcon, Paul Coppo, Julie Egea

**Affiliations:** ^1^ Division of Medical Oncology “CHR Metz‐Thionville” Ars‐Laquenexy France; ^2^ Nephrology Dialysis and Apheresis Unit “CHR Metz‐Thionville” Ars‐Laquenexy France; ^3^ Department of Pathology “CHR Metz‐Thionville” Ars‐Laquenexy France; ^4^ Department of Haematology Reference Centre for Thrombotic Microangiopathies, Saint‐Antoine University Hospital, Assistance Publique‐Hôpitaux de Paris Paris France

**Keywords:** hematology, nephrology, oncology, pathology and laboratory medicine, pharmacology and pharmacy

## Abstract

Paraneoplastic (p) TTP is a rare syndrome characterized by an immune‐induced, generalized microangiopathy associated with solid or hematological tumors. This case, reporting a patient with a metastatic HER2+ breast cancer and a pTTP, highlights the rarity of this entity, its difficult and challenging diagnosis, and the complexity of its management.

## 1 Introduction

Thrombotic microangiopathy (TMA) is characterized by a generalized microvascular occlusion by platelet thrombi, leading to mechanical hemolytic anemia (HA), thrombocytopenia, and organ failure [1, 2]. TMA includes thrombotic thrombocytopenic purpura (TTP), which is related to a severe deficiency in the von Willebrand factor (VWF)—cleaving metalloproteinase ADAMTS13 (VWF disintegrin and metalloprotease with thrombospondin type 1 repeats, member 13), either idiopathic (iTTP) or congenital (cTTP), hemolytic and uremic syndrome (HUS), with a predominant renal involvement [3, 4, 5], including typical HUS, caused by bacterial infections secreting shiga toxin, and atypical (a) HUS, associated with coagulation or complement alternative pathway dysregulation, and drug‐induced (DI) TMA, including patients previously reported as having both DI‐HUS and DI‐TTP [3, 4].

In our case, a metastatic breast cancer (BC) patient, receiving a gemcitabine/trastuzumab combined chemotherapy, presented with asthenia, anorexia, thrombocytopenia, microcytic, normochromic anemia, and severe renal failure. A diagnosis of paraneoplastic TTP (pTTP) was made as we found a low ADAMTS‐13 activity (< 10%) and the presence of anti‐ADAMTS‐13 IgG antibodies, both being very infrequent or absent in gemcitabine‐induced (G)‐TMA [6, 7, 8, 9, 10, 11, 12]. The patient underwent dialysis and several plasma exchanges (PEX) without any substantial clinical or biological improvement. A new chemotherapy was started, but the patient died 2 months later because of a tumor progression.

### 1.1 Case History, Examination, and Presentation

**FIGURE 1:**
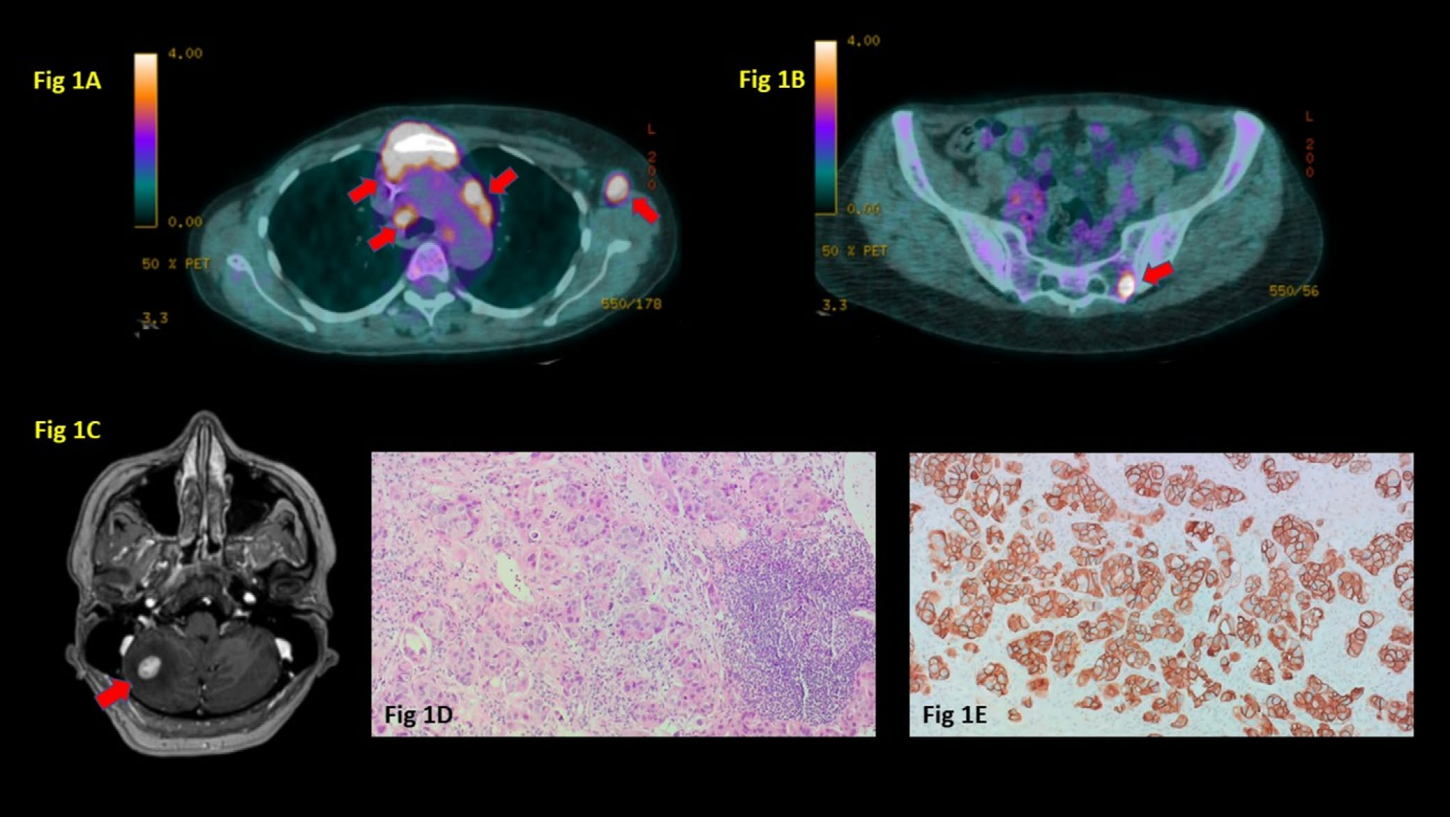
Diagnosis of metastatic breast cancer. (A) Hypermetabolic, sternal and mediastinal lymph node metastases (red arrow; thoracic section, ^18^fluoro‐2‐deoxy‐D‐glucose (FDG)‐positron emission tomography (PET) scan). (B): Hypermetabolic bone metastasis (red arrow; pelvic axial section PET scan). (C) Cerebellar metastasis with perilesional oedema (red arrow; axial section, magnetic resonance image (MRI). (D) Lymph node metastasis from a breast cancer (histology; hematoxylin and eosin staining, 100×). (E) Positivity of tumor cells for HER2 (immunohistochemistry; hematoxylin and eosin staining, 200×).

In June 2021, a 53‐year‐old Armenian non‐smoker woman with a metastatic HER2+ BC was hospitalized for asthenia, anorexia, a grade 3 thrombocytopenia, a grade 2 microcytic normochromic anemia, and a severe renal failure. She had a moderate chronic renal failure as a relevant comorbidity. In 2012, the patient underwent in Armenia a right mastectomy with sentinel lymphadenectomy for an early BC (initial tumor stage unknown), followed by adjuvant chemotherapy (protocol unknown). In 2015, she presented a local breast relapse needing a radical mastectomy with axillary lymph node dissection, followed by a post‐surgical chemotherapy with a carboplatin/paclitaxel regimen and a hormonotherapy with letrozole. In 2016, she went to France. The ^18^fluoro‐2‐deoxy‐D‐glucose (FDG)‐positron emission tomography (PET) scan documented multiple bone and lymph node metastases (Figure 1A,B). The magnetic resonance image (MRI) found a solitary cerebellar metastasis (Figure 1C) that was treated with total brain radiotherapy (dose of 39 Gy/13 fractions). The percutaneous lymph node biopsy confirmed the presence of atypic, massively infiltrating tumor cells with a pleomorphic and hyperchromic nucleous, forming glandular structures associated with several areas of necrosis (Figure 1D). At immunohistochemistry, tumor cells were positive for the human epidermal growth factor receptor 2 (HER2) (Figure 1E) and cytokeratin 7 and negative for hormonal receptors (HR), consistent with the diagnosis of a metastasis from a HR‐/HER2+ BC. The patient received a systemic chemotherapy with docetaxel and pertuzumab/trastuzumab until May 2018 when a second‐line treatment with ado‐trastuzumab emtansine was administered because of a tumor progression. In March 2020, we started a third‐line chemotherapy with oral vinorelbine and trastuzumab followed, in February 2021, by a fourth‐line treatment with gemcitabine and trastuzumab for another tumor progression. The patient received a cumulative dose of gemcitabine of 11.150 mg. The patient's performance status (ECOG) was 2. Clinical examination revealed irregular hepatomegaly at 4 cm and important bilateral oedemas of the legs.

### 1.2 Differential Diagnosis, Investigations, and Treatment

Biological tests showed a microcytic, hypochromic anemia at 8.3 g/dL (11.5 g/dL < NV > 16 g/dL) associated with the presence of schistocytes, low levels of haptoglobin, a thrombocytopenia at 39 × 10^9^/L (150 × 10^9^/L < NV > 450 × 10^9^/dL), high levels of creatinine at 293 μmol/L (45 μmol/L < NV > 90 μmol/L) (glomerular filtration rate: 15 mL/min), and a hypoalbuminemia at 26 g/L (35 g/L < NV > 52 g/L). The direct Coombs test was negative. The bilirubin levels as well as the free hemoglobin and lipids were in the normal range. Based on the clinical history and the biological analysis, after a multidisciplinary team discussion, we firstly hypothesized a diagnosis of a G‐TMA as the renal symptoms were at the foreground, few or no neurological symptoms were observed, and the disease occurred after the last gemcitabine infusion. At this time, only hemodialysis was performed since the patient became anuric with marked oedemas. However, 3 weeks later, considering the persistent severe thrombocytopenia and the absence of relief despite the gemcitabine interruption, we evoked the diagnosis of a pTTP. The renal biopsy was not performed because of the severe thrombocytopenia. The plasmatic activity of ADAMTS‐13 was very low (< 5%, 50% < NV < 150%) and was associated with the presence of anti‐ADAMTS‐13 IgG (34 IU/mL, NV < 25 IU/mL) confirming the hypothesis of a pTTP. During the hospitalization, the patient's clinical conditions worsened, and she presented an acute cardiac failure with a left ventricular ejection fraction (LVEF) of 35% and a plasmatic level of natriuretic peptide B at 2363 ng/mL (NV < 100 ng/mL) needing oxygen therapy and the administration of diuretics. The patient underwent corticosteroids, multiple red cell and platelet transfusions coupled with a dialysis, performed two to three times weekly, and 8 PEX treatments every 1 to 2 days, with 100% of fresh frozen plasma replacement without any biological or clinical improvement. After this treatment, the plasmatic ADAMTS‐13 levels were always low (< 5%, 50% < NV < 150%). As the patient presented a metastatic and poor prognosis BC progressing after several lines of chemotherapy and her clinical conditions were stable, we did not consider beneficial a treatment by rituximab or caplacizumab; nevertheless, we started a new systemic chemotherapy by a weekly paclitaxel and trastuzumab regimen.

### 1.3 Outcome and Follow‐Up

A cardiac MRI, performed a month later, showed an improvement of the LVEF at 47% without any myocardial fibrosis. Two months later, the patient died due to rapid tumor progression.

## 2 Discussion

Firstly, described in 1924 by Moschcowitz [1], TMA includes several clinical/biological entities such as congenital (c) and idiopathic (i) TTP, typical HUS, atypical (a) HUS, associated with coagulation or complement‐mediated mechanisms, and drug‐induced (DI)‐TMA [3, 13, 14, 15]. TTP, typically characterized by a deficit in the activity (< 10%) of ADAMTS13 leading to the accumulation of ultra‐large VWF multimers platelet aggregation, endothelial damage, and microvascular thrombosis and ischemia, shows an incidence of < 1/100 000/year [13, 14, 15]. TTP is caused by inherited mutations in ADAMTS13 (cTTP) or, more frequently, by the acquired presence of autoantibodies inhibiting the plasma ADAMTS13 activity (immune‐induced TTP) [6, 16, 17]. Typical HUS is usually associated with bacterial infections producing Shiga toxin, particularly *Escherichia coli* O157:H7 [15, 16]. The dysfunction of the coagulation (thrombomodulin, plasminogen, or diacylglycerol kinase epsilon) or the complement alternative pathway (approximately 60% of cases involving genes encoding complement regulators, such as factor H, membrane cofactor protein CD 46, and factor I, or complement activators, such as factor B and complement component C3) has been reported in aHUS [6, 15, 16, 17]. Finally, DI‐TMA is associated with drug exposure, including clopidogrel, calcineurin inhibitors, estrogen/progesterone, gemcitabine, interferon, mitomycin, cisplatin, bleomycin, 5‐fluorouracil, quinine, ticlopidine, oxymorphone, bevacizumab, carfilzomib, ixazomib, palbociclib, herbal remedies, and illicit drugs and includes patients previously reported as having both DI‐HUS and DI‐TTP [6, 15, 16, 17]. Two potential mechanisms for DI‐TMA have been proposed: immune‐mediated reactions and dose or duration‐related toxicity [15]. However, unlike immune‐induced TTP, DI‐TMA is rarely associated with severe deficiency of ADAMTS13 levels or presence of inhibitors [6, 15, 16, 17]. Its pathogenesis is usually associated with autoimmunity, direct endothelial toxicity, and drug‐dependent antibodies [6, 7, 8, 15]. The causal relationship between a specific anticancer therapy and TMA is difficult to establish as the tumor itself can induce TMA, and cancer patients often receive several concomitant drugs [9]. The differential diagnosis between a G‐TMA, defined by at least two of the three following criteria: (a) HA, thrombocytopenia, and acute kidney injury (AKI) or (b) a biopsy‐proven renal TMA [10, 11, 12] and a pTTP is often challenging in cancer patients, particularly in a context of a metastatic setting, as the role of gemcitabine in patients with a progressive tumor, frequently receiving many concomitant chemotherapies, is difficult to define [10, 11]. However, in G‐TMA, the ADAMTS13 levels are typically normal [9, 15]. It is also interesting to note that pTTP is usually characterized by a predominant kidney failure, unlike the other immune‐induced TTP subtypes where neurological and cardiac symptoms are more frequent [16, 17]. In any case, a very low platelet count (< 20 × 10^9^/L) should draw attention to the probability of an immune‐induced TTP using the French Score [16, 17].

Clinically, TMA presents a Coombs‐negative HA with elevated serum lactate dehydrogenase (LDH) level, undetectable or markedly decrease serum haptoglobin and the presence of schistocytes, a thrombocytopenia, an AKI, a proteinuria or a hypertension, neurologic and gastrointestinal symptoms and a normal coagulation [1, 2, 3, 4, 6, 15, 16, 17]. Histologically, the renal biopsy often documents eosinophilic hyaline thrombi, mainly composed by platelet aggregates [1, 2, 3, 4].

Recently, the French registry reported a prevalence of TTP of 13 cases per million people [7]. At presentation, 378 (49%) patients had idiopathic TTP, whereas 394 (51%) patients presented a disease associated with (infections, autoimmunity, pregnancy, cancer, and organ transplantation) or a drug administration. Three distinct forms of TTP were observed, including an autoimmune disease with anti‐ADAMTS13 IgG (75%), an acquired disease of unknown origin (22%) and an inherited disease (Upshaw–Schulman syndrome) with mutations of the *ADAMTS13* gene (3%) [7].

The level of ADAMTS13 activity defining a severe deficiency remains uncertain, recent data suggesting that ADAMTS13 activity < 10% alone could be consistent with the diagnosis and treatment of TTP [8]. However, several observations did not confirm this value [8]. Measurements of the ADAMTS13 activity may not accurately reflect the in vivo ADAMTS13 function as high levels of bilirubin may cause the report of a falsely low activity by interfering with the biological techniques and, on the contrary, the dissociation of the anti‐ADAMTS13 IgG from the ADAMTS13 during the in vitro incubation may determine a falsely high activity [8]. These data clearly support the importance of the patient's clinical symptoms in the diagnosis of TTP.

As the pTTP is usually an autoimmune disorder, corticosteroids are considered a standard treatment [18, 19, 20, 21, 22]. Since 2002, several data support the use of rituximab as routine initial treatment together with PEX and corticosteroids [23, 24, 25]. This drug, providing additional immunosuppression, decreased the frequency of relapse without modifying the prognosis [17, 23, 24, 25]. Recently, many other treatments are available with a controversial activity, including caplacizumab, a humanized, bivalent nanobody targeting the A1 domain of vWF to inhibit the interaction between vWF and platelets [17, 26, 27], and recombinant ADAMTS13 [17, 28].

## 3 Conclusions

pTTP is a very uncommon and complex syndrome that is usually associated with a poor prognosis. The diagnosis, based on clinical and biological features, is difficult to make and represents a clinical challenge as well as its multidisciplinary management.

## Author Contributions

**Raffaele Longo:** conceptualization, data curation, formal analysis, funding acquisition, investigation, methodology, project administration, resources, supervision, validation, visualization, writing – original draft, writing – review and editing. **Benjamin Savenkoff:** conceptualization, formal analysis, funding acquisition, investigation, supervision, validation, writing – original draft, writing – review and editing. **Romane Ribal:** data curation, formal analysis, validation, writing – original draft, writing – review and editing. **Alexandre Dadoun:** data curation, investigation, validation, writing – original draft, writing – review and editing. **Marco Campitiello:** data curation, methodology, validation, writing – original draft. **Francesca Plastino:** data curation, formal analysis, validation, writing – original draft. **Pierre‐Olivier Legros:** data curation, investigation, validation, writing – original draft. **Nathalie Marcon:** data curation, investigation, validation, writing – original draft, writing – review and editing. **Paul Coppo:** data curation, investigation, methodology, supervision, validation, writing – original draft, writing – review and editing. **Julie Egea:** data curation, validation, writing – original draft, writing – review and editing.

## Ethics Statement

The authors have nothing to report.

## Consent

Written informed consent was obtained from the patient for publication of this case report and any accompanying images. A copy of the written consent is available for review by the *Editor‐in‐Chief* of the journal.

## Conflicts of Interest

The authors declare no conflicts of interest.

## Data Availability

All data and materials are available for review at the Division of Medical Oncology, CHR Metz‐Thionville, in an electronic format.
